# P-858. Impact of Infectious Diseases Consultation on the Management of Staphylococcus Aureus Bacteremia in a Large, Predominately Rural, Health System

**DOI:** 10.1093/ofid/ofae631.1050

**Published:** 2025-01-29

**Authors:** Muhammad Murtaza, Catessa A Howard, Jesse Thompson, Michael B Edmond, Michael Stevens

**Affiliations:** West Virginia University SOM and Hospitals, Morgan Town, West Virginia; West Virginia University Medicine, Morgantown, West Virginia; West Virginia University School of Medicine, MorganTown, West Virginia; West Virginia University, Morgantown, West Virginia; West Virginia University, Morgantown, West Virginia

## Abstract

**Background:**

Staphylococcus Aureus Bacteremia(SAB) is associated with high morbidity and mortality. Infectious Diseases (ID)consultation has been associated with a decrease in mortality. The WVU Medicine Health System (WVUM) serves as a predominantly rural population and includes 24 hospitals (9 of which are critical access hospitals). Access to ID consultations is limited across the system and region.

**Methods:**

We generated a list of patients with SAB who presented to WVUM hospitals between 6/1/22 to 5/30/2023. A subset of patient charts were selected for review. We manually reviewed patient records and recorded key information and assessed whether patients met IDSA guidelines criteria for complicated versus uncomplicated SAB.

**Results:**

Please see Table 1

**Table 1.** Management of SAB for Patients With and Without ID Consultation

Variable	Patients with ID Consult (143)	Patients without ID Consult (93)	P-value
Diagnostic Imaging	MRI C-spine: 27/143 (19%)	MRI C-spine: 2/93 (2%)	< 0.001
	MRI T-spine: 32/143 (22%)	MRI T-spine: 4/93 (4%)	< 0.001
	MRI LS spine: 41/143 (29%)	MRI LS spine: 3/93 (3%)	< 0.001
	TTE: 129/143 (90%)	TTE: 38/93 (41%)	< 0.001
	TEE: 62/143 (43%)	TEE: 3/93 (3%)	< 0.001
Metastatic Infection	48/143 (34%)	5/93 (5%)	< 0.001
Complicated	139/143 (97%)	93/93 (100%)	0.1557
Antibiotic duration	< 2 weeks: 0/143 (0%)	< 2 weeks: 52/93 (56%)	< 0.001
	2 weeks 10/143 (7%)	2 weeks 16/93 (17%)	0.025
	4 weeks 24/143 (17%)	4 weeks 2/93 (2%)	< 0.001
	6 weeks 95/143 (66%)	6 weeks 10/93 (11%)	< 0.001
Died within 72 hours of presentation	0/143 (0%)	16/93 (17%)	< 0.001
30 Day re-admission*	30/143 (21%)	26/77 (34%)	0.301
30-Day mortality*	10/143 (7%)	11/77 (14%)	< 0.001

*Excluding people who died within 72 hours of presentation

**Conclusion:**

We found major differences in the work up of SAB and in clinical outcomes for patients receiving ID consultation and those who did not. Patients who received ID consultation were more likely to receive guidelines compliant evaluation and treatment, and were less likely to be re-admitted and die. When patients who died within 72 hours of presentation were excluded from analysis , patients without ID consults were still significantly more likely to die. These data will inform efforts to expand access to ID consultation for patients with SAB across our health system.

**Disclosures:**

**All Authors**: No reported disclosures

